# Novel Insight into the Formation of Odour—Active Compounds in Sea Buckthorn Wine and Distilled Liquor Based on GC–MS and E–Nose Analysis

**DOI:** 10.3390/foods11203273

**Published:** 2022-10-20

**Authors:** Yanan Xia, Musu Zha, Hao Liu, Quan Shuang, Yongfu Chen, Xujin Yang

**Affiliations:** 1 Key Laboratory of Dairy Biotechnology and Engineering, Ministry of Education, Inner Mongolia Agricultural University, Hohhot 010018, China; 2 College of Food Science and Engineering, Inner Mongolia Agricultural University, Hohhot 010018, China

**Keywords:** sea buckthorn, wine, VOCs, OAV, differential metabolites, differential pathways

## Abstract

Sea buckthorn wine (SW) and distilled liquor (DL) are fruit wines with beneficial health effects. However, their unpleasant flavour limits their development and widespread acceptance. Therefore, it is necessary to analyse their flavour composition and changes. In this study, differential metabolites of sea buckthorn DL during processing were analysed, and the relationships between E–nose sensor values and key volatile organic compounds (VOCs) were established. The results show that 133 VOCs were identified, with 22 aroma–contributing components. Fermentation significantly increased the content of VOCs, especially esters. A total of seven and 51 VOCs were significantly upregulated after fermentation and distillation, respectively. Meanwhile, seven sensors were positively correlated with the increased level of alcohols and esters, and reflected the increasing trends of 10 key VOCs.

## 1. Introduction

Sea buckthorn (*Hippophae rhamnoides*), classified as a medicine food homology (MFH) plant, contains large amounts of vitamin C, carotene, flavones, linoleic acid and other bioactive substances [1,2]. For example, the vitamin C content of sea buckthorn juice (SJ) reaches 1000 mg/100 g, which is two to three times higher than that of kiwifruit (*Actinidia deliciosa*) [3]. Sea buckthorn has attracted a great deal of attention in recent years due to the functions of preventing blood stasis, strengthening the spleen and stomach and even fighting against cancer [4]. However, the sour taste existing in sea buckthorn has resulted in its application being confined to the products of juice, yogurt and oil.

Sea buckthorn wine is obtained by fermentation of SJ with yeast or koji after acid reduction and sugar adjustment. Negi [5] developed sea buckthorn wine with significant antioxidant activity and higher levels of flavonoids, quercetin, waxberry and rutin compared to Sauvignon salad wine. The report also showed that it had a protective effect against the oxidative stress response induced by furone and high–cholesterol diet–induced hypercholesterolemia in male mice of the LACA strain. However, the industrial development of this potentially beneficial product is limited because of its unpleasant flavour. Therefore, it is necessary to analyse the flavour of SW and sea buckthorn distilled liquor (DL).

Many methods and instruments are available for the determination of volatile organic compounds (VOCs) in food, including gas chromatography (GC), gas chromatography–mass spectrometry (GC–MS), electronic nose (E–nose), electronic tongue (E–tongue), etc. VOCs in food have complex compositions and uneven distributions, making it difficult to elucidate flavour information using a single analytical technique [6,7]. GC–MS is one of the most commonly used methods for food flavour analysis because GC can effectively separate complex substances and allows relatively accurate quantitative analysis, while MS allows qualitative analysis of component substances [8,9]. For example, the most abundant derivatives of sea buckthorn from hilly areas of the Romanian Eastern Carpathians were ethyl esters of 2–methylbutanoic acid, 3–methylbutanoic acid, hexanoic acid, octanoic acid and butanoic acid, as well as 3–methylbutyl 3–methylbutanoate, 3–methylbutyl 2–methylbutanoate and benzoic acid ethyl ester [10]. The volatile compounds extracted from the leaves of 14 *H. rhamnoides* populations from China were analysed by GC–MS and 44 compounds were detected, and the main compounds included tetracosane (10–40%), hexadecanoic acid (<0.1–11%), octadecatrienol (5–27%), tetracosene (3–11%), eicosanol (<0.1–13%) and others [11]. However, the threshold value of each aroma substance is different, and it is not enough to evaluate its contribution to the overall flavour only by its content. Combined with the flavour threshold, the odour active value (OAV) can be calculated to determine the components that contribute to the flavours of foods [12]. Furthermore, based on a metabolomics approach, screening for differential metabolites before and after treatment can be used to annotate relevant metabolic pathways via Kyoto Encyclopedia of Genes and Genomes (KEGG) database analysis, and further determine the mechanisms of VOC formation [13,14,15].

E–nose is an instrument used for the rapid detection of VOCs in food. The E–nose sensor converts the smells of odour substances into electrical signals, and the responses of multiple sensors constitute the response spectrum of the sensor array to a given smell. Different odours can be distinguished by their characteristic response spectrum [16]. At present, electronic nose is not widely used. It is mainly used for the difference analysis or authenticity identification of food from different sources or craft. Liu et al. [17] found that there was a difference in the volatile substances among the different LAB–fermented sea buckthorn juice by E–nose; the juice fermented by *Lactobacillus plantarum* LP56 (SBJLp) could be easily identified by sensors sensitive to aromatic substances. Its advantage is that it is fast and convenient, but its disadvantage is that the sensor response value cannot accurately reflect the content of a specific substance. Conversely, GC–MS metabolome analysis is good at qualitative and quantitative analysis of flavour compounds. Therefore, correlation analysis between key VOCs identified by GC–MS metabolomics and E–nose data is useful, as the changes in key VOCs can be quickly verified based on the corresponding values of specific sensors [18].

Here, we compared changes in the flavour of sea buckthorn DL among different stages of processing, analysed the flavour profiles of SW and its DL, identified differential metabolites and key metabolic pathways, and then established the relationships between E–nose data and key VOCs to provide a basis for flavour optimisation of SW.

## 2. Materials and Methods

### 2.1. Production of SW and DL

**Figure 1:**
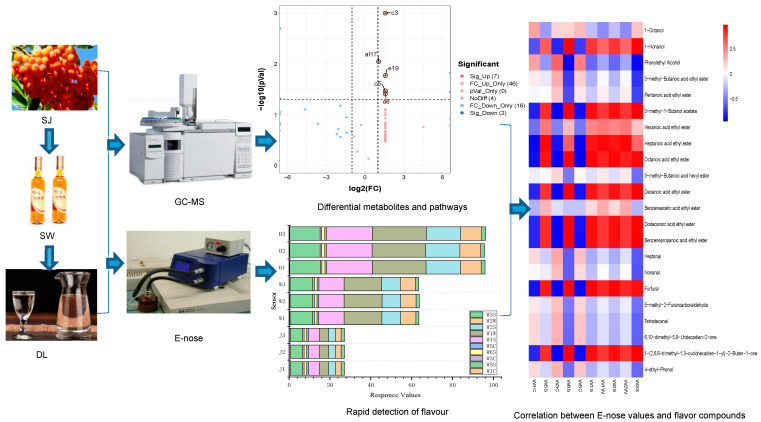
The whole research process.

According to the method of He [19], frozen sea buckthorn berries were thawed in the shade for 24 h at 4 °C, and cleaned with tap water. Then, the berries were pulped with a JYZ–E19 slow juicer (Jiuyang Co., Jinan, China) at 20,000 rpm for 1 min and depectinised by pectinase (1 g/L, about 0.05%) (Novozymes Co., Tianjin, China) at room temperature for 3 h. The juice (SJ1, SJ2, SJ3) was then adjusted to pH 3.7 and 20 °Bé soluble sugar with NaHCO_3_ and glucose, respectively (soluble sugar content was measured by MC202231 handheld sugar meter (*Chengdu Taiguang Learning Co.,* Ltd., Chengdu, China). Additionally, 30 mg/L potassium metabisulfite was added to SJ to sterilise and improve stability. The samples were inoculated with 0.2% (*w/v*) *Saccharomyces cerevisiae* (Angel Yeast Co. Ltd., Yichang, China) that had been activated in 2% glucose solution at 37 °C (water bath) for 30 min, and then underwent fermentation at 28 °C for 7 days (the CO_2_ produced by fermentation can be discharged into the water with the hose) to obtain SW (SW1–SW3) with an alcohol content of 13  ±  2% (*v*/*v*). Finally, 1L SW was distilled, producing 500 mL distillate for the first time (GG–17 all–glass distiller, Guangzhou Diangrui Glass Experimental Instrument Co., Ltd., Guangzhou, China), and 250 mL distillate liquor (DL1–DL3) with an alcohol content of 40  ±  2% (*v*/*v*) was obtained by second distillation. The whole research process is shown in Figure 1.

### 2.2. Determination of Antioxidant Activity

#### 2.2.1. OH· Radical Scavenging Rate

Based on the method of Xu et al. [20], 1 mL samples were obtained and made up to 2 mL with distilled water (diluted samples). To the tubes containing 2 mL of diluted sample, we successively added 2 mL of 6 mmol/L FeSO_4_ solution and 2 mL of 6 mmol/L H_2_O_2_ solution, followed by shaking. The mixture was allowed to stand for 10 min at room temperature. Then, 2 mL of 6 mmol/L salicylic acid was added and allowed to stand for 30 min at room temperature. Each sample was determined three times in parallel. Ascorbic acid was used as a positive control. The OH· radical scavenging rate was determined using the following formula:

OH· radical scavenging rate (%)  =  [1 −  (A_i_  − A_j_)/A_0_]  ×  100 (1)

where A_i_ is the absorbance of the sample at 510 nm, A_j_ is the absorbance measured after replacement of H_2_O_2_ with distilled water, and A_0_ is the absorbance measured in the blank control group with distilled water instead of the sample.

#### 2.2.2. DPPH Clearance Rate

According to the method of Liu, Ooi and Chang [21], 2 mL of 0.04 mg/mL 2,2–diphenyl–1–picrylhydrazyl (DPPH) solution was added to 2 mL of diluted sample, mixed, and allowed to react for 20 min. Each sample was determined three times in parallel. Ascorbic acid was used as a positive control. The DPPH clearance rate was determined using the following formula:

DPPH clearance rate (%)  =  [1 −  (A_i_  −  A_j_)/A_0_]  ×  100 (2)

where A_i_ is the absorbance of the supernatant at 517 nm, A_j_ is the absorbance measured after replacement of DPPH solution with anhydrous ethanol, and A_0_ is the absorbance of the reference consisting of 2 mL 0.04 mg/mL DPPH and 2 mL of anhydrous ethanol reactant.

#### 2.2.3. Reducing Activity

According to the method of Kaewnarin et al. [22], 1 mL samples were taken and made up to 2.5 mL with distilled water, followed by the addition of 2.5 mL of 0.2 mol/L phosphoric acid buffer (pH 6.6) and 2.5 mL of 1% potassium ferric cyanide solution. After incubation at 50 °C in a water bath for 20 min, the samples were rapidly cooled. Then, 2.5 mL of 10% trichloroacetic acid was added and centrifuged at 3000 rpm for 10 min. After centrifugation, 5 mL of the supernatant was taken, and 4 mL of distilled water and 1 mL of 0.1% ferric chloride solution were added and mixed by oscillation. The absorbance at 700 nm was measured after 10 min. Ascorbic acid was used as a positive control. Each sample was determined three times in parallel.

### 2.3. GC–MS Detection

According to the method of Xia et al. [23], with minor modifications, 0.5 g of NaCl and 100 ppm 3–octanol (internal standard substance) was added to 10 mL of sample solution in 20 mL sealed glass vials. The samples were extracted at 40 °C for 40 min with 50/30 μm divinylbenzene/carboxen/polydimethylsiloxane (DVB/CAR/PDMS) fibre (Supelco, Bellefonte, PA, USA). Flavour compounds were detected by GC–MS (5975 mass spectrometer coupled to a 7890A gas chromatograph; Agilent, Santa Clara, CA, USA). A DB–INNOWax column (60 m  ×  0.25 mm ID and 0.25 μm film thickness) was used for separation. The temperatures of the injector, electron ionisation source, quadrupole chamber and transfer lines were 250 °C, 230 °C and 250 °C, respectively. The initial temperature was 50 °C for 3 min, which was increased to 80 °C at a rate of 3 °C/min. The temperature was further increased to 230 °C at 5 °C/min and maintained at 230 °C for 6 min. The carrier gas had a flow rate of 1.0 mL/min. Samples were injected in splitless mode. A mass range of 50–550 *m*/*z* was recorded at a rate of one scan per second. Each sample was determined three times in parallel.

Data were analysed using GC–MS software (Agilent, CA, USA) and identified based on the NIST 2017 database. Only compounds with a matching degree ≥80 were retrieved and recorded. The NIST database was used to automatically retrieve mass spectral data of each component for qualitative analysis. A semiquantitative method was used to determine relative contents by calculating the ratio of the internal standard substance (3–octanol) to the peak area of each component. Principal component analysis was used to analyse the differences in the flavour of the compounds among the three types of samples (SJ, SW and DL). Odour activity value (OAV) was obtained by the ratio of the concentration of the substance to the threshold value.

### 2.4. E–Nose Measurement

In accordance with the method of Cui et al. [24], E–nose (PEN3, German AIRSENCE company, Schwerin, German) was applied with a detection time of 120 s, cleaning time of 80 s, pre–injection time of 5 s, injection flow rate of 400 mL/min, and carrier gas (clean air) flow rate of 400 mL/min. At the beginning of measurement, the sensor fluctuated with time and began to flatten after 110 s. The sample was diluted 1:40, and 10 mL was then added to a test tube (20 mL), which was subsequently sealed with plastic wrap. After 30 min in a water bath at 50 °C, headspace gas of the samples was analysed by E–nose. Data at 114, 115 and 116 s were used to calculate the average for analysis. Each sample was determined three times in parallel. The 10 sensor values corresponding to nine samples (S1–SJ3, SW1–SW3, DL1–DL3) were used for stacking bar chart (Origin 9.0 software, OriginLab Co., Northampton, NC, USA). Principal component analysis was used to analyse the differences of the overall odour among the three groups of samples (SIMACA–P software, Umetrics Co., Malmo, Sweden). Furthermore, the correlation analysis (R software, The University of Auckland, Auckland, New Zealand) between the response value of different sensors of electronic nose and the content of flavour substances in GC–MS was carried out to establish the relationships between E–nose data and key VOCs to provide a basis for the rapid detection and flavour optimisation of SW.

### 2.5. Statistical Analysis

SPSS17.0 software was used for variance analysis. A value of *p* < 0.05 represented significant difference, *p* < 0.01 represented extremely significant difference, and the experimental results were expressed as the mean ± standard deviation. Each index was repeated three times. Principal component analysis was performed using SIMCA–P software, line charts and bar charts were constructed in Origin 9, and R language was used for the generation of Venn diagrams, volcano diagrams, and correlation heat maps.

## 3. Results and Discussion

### 3.1. Physicochemical Characteristics of Sea Buckthorn Wine

**Figure 2:**
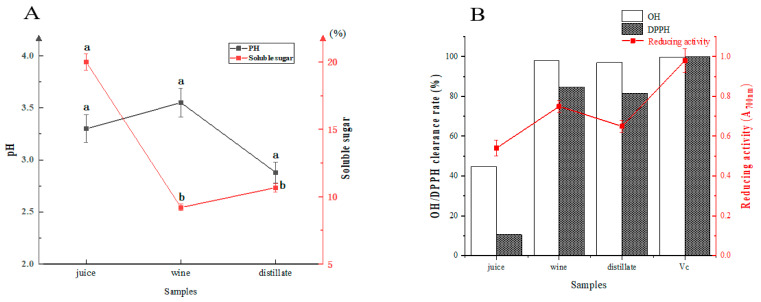
Comparison of the physicochemical indices (**A**) and antioxidant activities (**B**) of SJ, SW and DL (*n* = 3). Error bars represent the standard deviation (SD). a and b represent significant difference.

The physicochemical indices and antioxidant activities of SJ, SW and DL are shown in Figure 2. The amount of soluble sugar in SJ was significantly decreased after fermentation, but no significant difference was observed after distillation (Figure 2A). The pH of SJ remained between 3.30 and 3.55, with no significant difference throughout the whole process. Meanwhile, the antioxidant activity of SJ was significantly enhanced by fermentation. For SW, the DPPH and OH· free radical clearance rates were significantly increased from 80.64% to 91.55% and from 44.72% to 97.10%, respectively. The reducing activity also had a significantly increasing trend from 3.79 to 3.94. He [19] reported the same trends during the production of SW. With the extension of fermentation time, the DPPH clearance rate and reducing activity improved significantly, reaching the maximum values at 6–8 d. Increases in levels of phenols and flavonoids were also found in the early stage of fermentation, which might be related to the improvement in antioxidant capacity. Wang et al. [25] found that at the concentration of 0.1 mg/mL, the DPPH radical scavenging activity of sea buckthorn tea leaves was 93.42%, indicating that sea buckthorn berry products have outstanding antioxidant activity. Nevertheless, distillation did not significantly decrease the antioxidant capacity (Figure 2B). Wang and Li [26] also found that when the volume of aloe liquor was more than 2.5 mL, the scavenging rate of ·OH could reach more than 80%.

### 3.2. Comparison of VOCs

**Figure 3:**
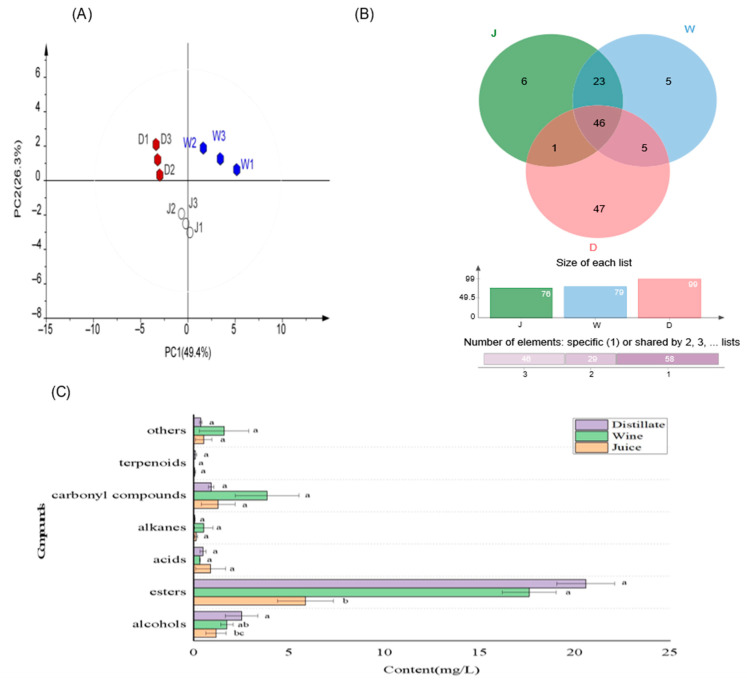
Comparison of VOCs of SJ, SW and DL by GC–MS (*n* = 3). (**A**) PCA analysis, (**B**) Venn diagram, (**C**) bar chart for comparison of various types of VOCs. Error bars represent the standard deviation (SD). a, b and c represent significant difference.

The comparison of VOCs of SJ, SW and DL is shown in Figure 3. PCA analysis showed that the variance explained by the first principal component (PC1) and second principal component (PC2) reached 75.7%, thus strongly reflecting the differences between samples [27]. The three groups of samples were distinctly separated from each other (Figure 3A), indicating significant differences in VOCs among the three groups. A total of 133 VOCs were detected by GC–MS, with 76, 79 and 99 identified in SJ, SW and DL, respectively (Figure 3B). Fermentation significantly increased the total content of VOCs, especially the ester components (Figure 3C). Esters were the most abundant components in all three groups. Their contents were increased about three to four fold by fermentation, followed by alcohols and carbonyl compounds (aldehydes and ketones). Xing [28] also reported that esters were increased by 13.03% in SW after fermentation and dominant (87.37%) in flavour compounds, which improved the fruit and ester aroma of SW and promoted flavour maturation.

Six types of flavour compounds were detected, only in SJ, and disappeared after fermentation; these compounds were β–copaene (t1), α–murolene (t3), oxaloacetic acid (ac6), *n*–decanoic acid (ac8), tridecane (alk3), and 5–tetradecene (alk7) (Figure 3B). Fermentation produced 10 new VOCs, including benzyl alcohol (al16), 1,1–dimethyl–cyclopropane (alk2), 3–ethyl–tridecane (alk5), styrene (alk4), 1–heptanol (al6), and 3–ethoxy–1–propanol (al5). SJ and SW shared 69 VOCs. Distillation promoted the output of a large number of VOCs in SW and 47 new flavour compounds were formed. Benzyl alcohol (al16), 1,1–dimethyl–cyclopropane (alk2), 3–ethyl–tridecane (alk5), styrene (alk4) and 2,3–butanediol (al8) were only present in SW, but were not detected after distillation.

### 3.3. Odour–Active Compounds

**Figure 4:**
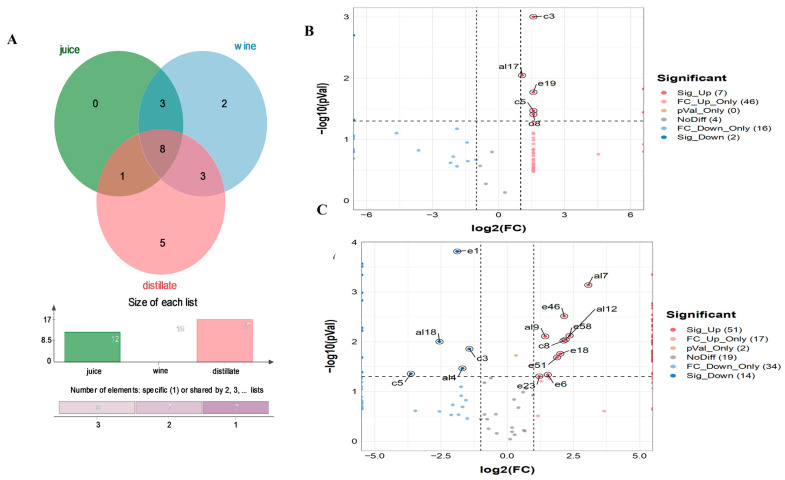
Odour–active compounds and differential metabolites in SJ, SW and DL by GC–MS (*n* = 3). (**A**) Venn diagram of odour–active compounds. Volcano plot of metabolites after fermentation (**B**) and distillation (**C**).

OAV is an index reflecting the contribution of one aroma to the overall flavour of samples. Table 1 lists the OAV values of VOCs in three groups of samples. There were 12, 16 and 17 odour–active components in SJ, SW and DL, respectively (Figure 4A). An aroma component with OAV > 1 can be considered as a contributor to the overall flavour, and a component with OAV  >  10 can be considered important [29]. The important VOCs of SJ included octanoic acid ethyl ester (OAV 1370.000), 3–methyl–butanoic acid hexyl ester (OAV 266.667), hexanoic acid ethyl ester (OAV 206.000), 1–nonanol (OAV 60.000) and 3–methyl–butanoic acid ethyl ester (OAV 18.723). Other VOCs contributing to the aroma were phenylethyl alcohol (OAV 9.956), 4–ethylphenol (OAV 8.467), nonanal (OAV 5.867), 1–octanol (OAV 1.815), pentanoic acid ethyl ester (OAV 1.624), tetradecanal (OAV 1.578) and 5–methyl––2–furancarboxaldehyde (OAV 1.060). According to the literature, the aromatic substances with OAV ≥1 in SBJLp (lactic acid bacteria)–fermented SJ included ethyl isovalerate and ethyl caproate [17]. Chauhan et al. [30] reported that the volatile aroma compounds of sea buckthorn berries were mainly aliphatic esters, such as ethyl esters of 3–methyl butyl and cis–3–hexen–1–yl. Esters usually have a fruity or floral odour, giving SJ a fresh and refreshing flavour [31,32].

Five VOCs increased in SW after fermentation, including benzeneacetic acid ethyl ester (OAV 2.610), geranyl acetone (6,10–dimethyl–5,9–undecadien–2–one, OAV 1.690), heptanal (OAV 1.500), heptanoic acid ethyl ester (OAV 1.182) and furfural (OAV 1.020). 4–Ethylphenol (OAV 25.400), phenylethyl alcohol (OAV 20.800) and nonanal (OAV 17.600) were added as important VOCs of SW. Phenylethanol with a rose fragrance is commonly found in wine, mangosteen and other fruit wines, and has been identified as a characteristic flavour compound. Ma [33] found that the relative content of phenylethanol in commercially sweet sea buckthorn wine was 7.193%. Lukša et al. [34] used *Hanseniaspora uvarum* yeast fermentation to produce sea buckthorn wine, and phenylethanol accounted for 3.6% of the overall aroma, indicating that phenylethanol is a stable flavour component in sea buckthorn wine. 4–Ethylphenol is usually present in rum and whiskey, and can be used as an essence in the preparation of wine and liquor [35,36]. Nonanal imparts a citrus fragrance (OAV 8.795) and contributes to the overall flavour of many wines [23].

DL also showed increased levels of five aroma components, i.e., β–damascenone (1–(2,6,6–trimethyl–1,3–cyclohexadien–1–yl)–2–buten–1–one, OAV 132,666.667), benzenepropanoic acid ethyl ester (OAV 488.889), decanoic acid ethyl ester (OAV 61.650), 3–methyl–1–butanol acetate (OAV 39.333) and dodecanoic acid ethyl ester (OAV 3.364); these were also characteristic VOCs of DL. It is worth noting that the contributions of 5–methyl–2–furancarboxaldehyde (OAV 0.300) and heptanal were significantly reduced in DL, while heptanal was not detected. β–Damascenone, which imparts berry and rose scents, is commonly detected in grape [37], acai berry and other fruits [38]. The β–damascenone in DL is derived from sea buckthorn. Another four esters that are also widely present in brandy and Chinese liquor [39] were the main flavour compounds of DL, and contributed greatly to the overall flavour. It has been reported that benzenepropanoic acid ethyl ester (OAV 18.69), decanoic acid ethyl ester (OAV 231.71), 3–methyl–1–butanol acetate (OAV 43.68) and dodecanoic acid ethyl ester (OAV 460.36) contribute to the aroma of jujube brandy [23].

### 3.4. Differential Metabolites

The flavour compounds with *p* < 0.05 and an FC value greater than 2 were selected as differential metabolites [40,41]. The flavour metabolites of SJ changed markedly after fermentation, with seven significantly upregulated and two downregulated VOCs (Figure 4B). Specifically, 3–octanone (c3), phenylethyl alcohol (al17), 2,3–butanediol (al8), tridecane (alk5), octanoic acid ethyl ester (e19), 6–methyl–5–hepten–2–one (c5) and acetoin (O8) showed an obvious increasing trend, while benzoic acid (ac10) and tridecane (alk3) obviously decreased. A total of 10 VOCs, including 2–methyl–propanol (al2), 3–ethoxy–1–propanol (al5), 1–heptanol (al6) and benzyl alcohol (al16), were newly generated in SW, along with significantly differential metabolites, and had log_2_FC values of infinity (Inf). 2,3–Butanediol imparts a sweet scent, and gave the wine a mellow and natural flavour. Seo et al. [42] also reported that 2,3–butanediol was a differential metabolite of ale and lager beers. 2–Methyl–propanol and benzyl alcohol, with unique alcohol/wine–like and floral scents, respectively, were produced in large quantities during fermentation, and they were also important to the overall flavour profile of fermented and distilled wines [23].

In total, 51 significantly upregulated VOCs were found in SW after distillation (Figure 4C), including 27 esters, eight carbonyl group compounds, four alcohols, three acids, two alkanes and seven other compounds. Linalool (al7), isobutyl isovalerate (e6), ethyl esters of octanoic acid (e18) and nonanoic acid (e23) showed obvious increasing trends. Linalool is naturally present in *Prunus dulcis* [43], tomato [44], tea [45] and kimchi [46], and not only gives food a lily fragrance, but also has antibacterial effects [47]. Overall, 14 downregulated VOCs were found in SW after distillation, including four alcohols (1–hexanol (al4), 2–furanmethanol (al13), benzyl alcohol (al6), phenylethyl alcohol (al7)), four esters (pentanoic acid ethyl ester (e1), butanoic acid, 1–methylpropyl ester (e3), hexanoic acid 1–methylethyl ester (e9), 3–methyl–butanoic acid ethyl ester (e15)), two carbonyl group compounds (6–methyl–5–hepten–2–one (c5), 3–octanone (c3)), acetic acid (ac1), tridecane (alk3) and acetoin (O8). The decrease in specific acids and alcohols is related to the formation of corresponding esters. For example, the decrease in acetic acid and phenylethanol is due to the esterification reaction of these two substances to further form phenylethyl acetate [48,49].

### 3.5. Rapid Detection of Flavour

**Figure 5:**
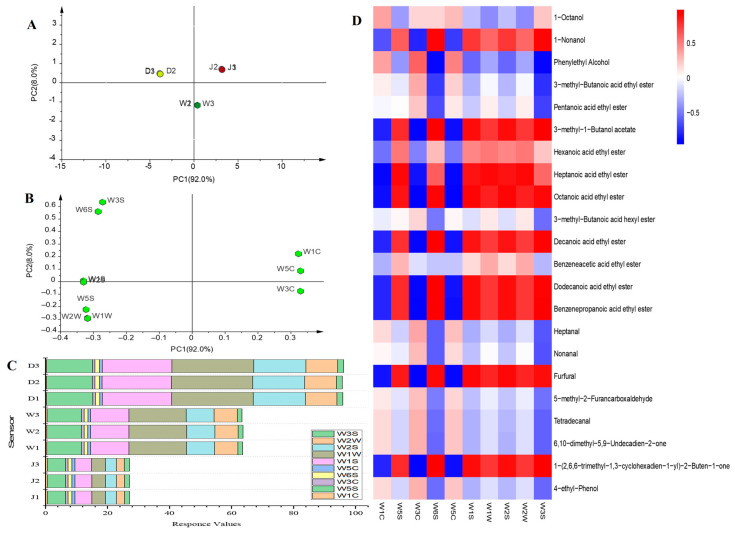
Comparison of flavour files in SJ, SW and DL by E–nose (*n* = 3). (**A**) PCA analysis, (**B**) load diagram of the sensors, (**C**) bar chart for response values of sensors, (**D**) correlation of sensors and key VOCs.

SJ, SW and DL samples were easily distinguished from each other by E–nose (Figure 5A). Fermentation and distillation significantly increased the response values of SW and DL (Figure 5B), indicating gradual enrichment of VOCs. The result is consistent with the findings of GC–MS. Correlation analysis was performed between the changes in E–nose values and each category of VOCs in the different groups (Figure 5C). The results indicate that seven sensors, W5S, W6S, W1S, W1W, W2S, W2W and W3S, were positively correlated with increased alcohol and ester contents. SBJLp (lactic acid bacteria)–fermented SJ also represented high response values on W1W, W1S, W5S and W3S, similar to this experiment result [17]. W1C, W3C and W5C were negatively correlated with alcohol and ester contents, and positively correlated with acid contents. W3C and W5C reflected the increasing trends of carbonyl compounds, alkanes and terpenoids. In comparison with SJ, the response values of W3S, W2W, W2S, W1W and W1S for SW and DL were significantly increased, indicating that the alcohols and esters in the samples increased significantly after fermentation and distillation (especially the former). This result is consistent with the trends detected by GC–MS. The above findings show that E–nose could be used for rapid detection and evaluation of the changing trends of the main flavour compounds observed during the production of SW and DL.

E–nose is not commonly used in flavour detection of sea buckthorn wine. Yu et al. [50] used E–nose to study the effects of different ultra–high–pressure treatments on the flavour and aging of SW, and found that treatment at 400 MPa significantly enhanced the aroma of SW. Some researchers have begun to establish correlations between GC–MS and E–nose data. For example, Long et al. [51] reported that S2, S6, S7 and S9 were important E–nose sensors for distinguishing between different cultivars of *Alpinia officinarum* based on the results of correlation analysis.

### 3.6. Correlation between E–Nose Values and Key Flavour Compounds

Correlation analysis was further conducted between the changes in response values of E–nose and 22 key flavour compounds (Figure 5D). W5S, W6S, W1S, W1W, W2S, W2W and W3S values were also found to be positively correlated with 10 key flavour compounds, including 1–nonanol (OAV 152.8), 3–methyl–1–Butanol acetate (OAV 39.3), hexanoic acid ethyl ester (OAV 670.0), heptanoic acid ethyl ester (OAV 1.6), octanoic acid ethyl ester (OAV 16,466.7), decanoic acid ethyl ester (OAV 61.7), dodecanoic acid ethyl ester (OAV 3.4), benzenepropanoic acid ethyl ester (OAV 488.9), furfural (OAV 4.4) and 1–(2,6,6–trimethyl–1,3–cyclohexadien–1–yl)–2–buten–1–one (OAV 132,666.7). W1C, W3C and W5C values were negatively correlated with the above 10 VOCs, indicating that these sensors reflected their downward trends. The above 10 VOCs all contributed to the overall flavour of sea buckthorn distilled liquor, particularly octanoic acid ethyl ester (OAV 16,466.7) and 1–(2,6,6–trimethyl–1,3–cyclohexadien–1–yl)–2–buten–1–one (OAV 132,666.7), indicating that the seven sensors (W5S, W6S, W1S, W1W, W2S, W2W and W3S) of E–nose can effectively reflect the strong aroma of sea buckthorn wine and distilled liquor in production. These results provide guidance for the rapid detection of the trends in key flavour compounds in SW and DL during production.

## 4. Discussion

### 4.1. Fewer Differential Metabolites before and after Fermentation

Compared with sea buckthorn juice, seven significantly upregulated substances were found, which, although seemingly small in number, were important aroma substances; these substances were 3–octanone, phenylethyl alcohol, 2,3–butanediol, tridecane, octanoic acid ethyl ester, 6–methyl–5–hepten–2–one and acetoin. In addition, there were in fact 10 important flavour compounds that were newly produced after fermentation in SW, including 2–methyl–propanol, 3–ethoxy–1–propanol, 1–heptanol, benzyl alcohol and so on. All these VOCs contributed greatly to the overall flavour of sea buckthorn wine.

The OAV of phenylethanol in sea buckthorn juice was 10, and the OAV of phenylethanol in sea buckthorn wine increased to 20.8, indicating that phenylethanol occupied an increasing proportion in the overall flavour of sea buckthorn wine with the fermentation. Phenylethanol is formed by phenylalanine transamination of phenylpyruvate, followed by decarboxylation to phenylaldehyde, and phenylaldehyde in the presence of oxidative dehydrogenase generates β–phenylethanol [48]. Phenylethanol has a rose aroma, and is a favourite aroma in many fruit wines. Ma [34] and Lukša et al. [34] found that the relative content of phenylethanol detected in sea buckthorn wine was 7.193% and 3.6%, respectively, indicating that phenylethanol is a stable flavour component in sea buckthorn wine.

The OAV of octanoic acid ethyl ester in sea buckthorn juice was 1370, and the OAV of octanoic acid ethyl ester in sea buckthorn wine increased to 4110, indicating that octanoic acid ethyl ester was one of the key flavour compounds of sea buckthorn juice and wine, and its contribution was more obvious after fermentation. Ma [33] and Lukša et al. [34] also found that the relative content of octanoic acid ethyl ester detected in sea buckthorn wine was 0.179% and 0.12%, respectively, indicating that octanoic acid ethyl ester is also a stable flavour component in sea buckthorn wine. In this experiment, the content of octanoic acid ethyl ester in sea buckthorn wine was 0.411 ± 0.114 mg/L. Although the content seemed low, the threshold value of octanoic acid ethyl ester was low as 0.1 ug/kg; therefore, its OAV value was very high, and it had a very high contribution to the overall flavour of sea buckthorn wine.

### 4.2. Metabolic Pathways before and after Fermentation

Seven significantly upregulated and two downregulated VOCs were found in SJ after fermentation (Figure 4B), and the metabolic pathways of fermentation were further analysed. In KEGG pathway analysis, the majority of metabolites were annotated as belonging to metabolic pathways and the biosynthesis of secondary metabolites (48 and 31 metabolites, respectively) (Figure S1A). After fermentation, 15 upregulated pathways were found (log_2_FC  >  1). The most obviously upregulated pathway was butanoate metabolism, followed by biosynthesis of cofactors, toluene degradation, furfural degradation, etc. (Figure S1B). Fermentation significantly downregulated the fatty acid biosynthesis and lipoic acid metabolism pathways. Variable importance in projection (VIP) values were calculated for pathways, including the VOCs newly generated during fermentation (log_2_FC = Inf), of which five had VIP values > 1 (Figure S1C): Butanoate metabolism, microbial metabolism in diverse environments, metabolic pathways, degradation of aromatic compounds and chemical–carcinogenesis DNA adducts.

Yang et al. [52] also found that the most relevant flavour pathway in baijiu is butanoate metabolism, which is related to *Thermoactinomyces*. Butanoate metabolism involves a total of 19 flavour substances, including two acids (3–methyl–butanoic acid, butanoic acid), three alcohols (2–butanol, 3–methyl–butanol, 2,3–butanediol) and 14 esters (3–methyl–1–butanol acetate, 3–methyl–butanoic acid ethyl ester, 3–methyl–1–butanol acetate, 3–methyl–butanoic acid hexyl ester, 1–methylpropyl ester, methyl–butanoic acid 2–methylpropyl ester, 2–methyl–butanoic acid 2–methylbutyl ester, etc.). The relevant differential metabolites included 3–methyl–butanoic acid, 2–butanol, 2,3–butanediol, 3–methyl–butanoic acid ethyl ester, 3–methyl–butanoic acid hexyl ester. These substances are important components of wine flavour. 3–Methyl butanoic acid was identified as a key aroma compound in fermented Forastero cocoa beans, and gave them a sweaty odour [53]. The specific markers of Luzhou–flavoured fresh raw liquor distilled from Zaopei were 2–butanol [54], 2,3–butanediol [55], butanoic acid, etc., indicating that 2–butanol is a common flavour component in specific fruit wines and distilled wines. 3–Methyl butanoate presents a fruity flavour, and is the main flavour ingredient of Chinese Premium famous Liquors [56] and Qingke Liquor [57].

Microbial metabolism in diverse environments and metabolic pathways are complex metabolic systems. Microbial metabolism in diverse environments includes carbohydrate metabolism, energy metabolism, metabolism of cofactors and vitamins and xenobiotic biodegradation. Metabolic pathways include carbohydrate metabolism, energy metabolism, lipid metabolism, nucleotide metabolism, amino acid metabolism, glycan metabolism, biosynthesis of terpenoids and polyketides, biosynthesis of other secondary metabolites and xenobiotic biodegradation.

### 4.3. Abundant Differential Metabolites before and after Distillation

Compared with the fermentation, the distillation process formed more flavour compounds. In total, 51 significantly upregulated and 14 downregulated VOCs were found in SW after distillation (Figure 4C). In the present study, 51 growing flavour compounds were of interest, including 27 esters, eight carbonyl group compounds, four alcohols, three acids, two alkanes and seven other compounds. Especially, key flavour compounds such as linalool, isobutyl isovalerate, ethyl esters of octanoic acid and nonanoic acid showed obvious increasing trends. Therefore, many scholars have studied the changes of flavour compounds in the process of liquor distillation, and further take this as a benchmark to effectively control the distillation process in production.

Yang et al. [58] found that distillates aroma compounds have significantly differences in time periods in five–grain liquor. Concentration of aldehydes, esters and aromatics were increased in phase 1–2 (1∼10 min), decreased in phase 3 (10∼15 min), and reached the stable level after phase 3. Concentrations of alcohols decreased with the distillation time. Concentrations of 6–methyl–5–hepten–2–one, furfural, 3–furanmethanol and vanillin increased firstly, then decreased, and increased after phase 3. Liu et al. [59] also found that during distillation process of Fen–flavour liquor in China, the contents of acetaldehyde, acetal, isovaleraldehyde, sec–butyl alcohol, isobutanol, ethyl acetate, ethyl butyrate, isoamyl acetate, ethyl caproate and 2–pentanone decreased gradually with the progress of distillation. The contents of acetochlor, ethyl lactate, ethyl palmitate, acetic acid and isobutyric acid increased gradually with the progress of distillation. The contents of acetal, methanol and ethyl linoleate in initial distillate were significantly higher than other fractions. Further sensory evaluation results show that the sensory quality of first–round liquor was better than second–round, and the sensory quality of the early fraction was better than that of the later fraction. These results all provide data support for the “pinching the initial distillate and removing the last distillate” in actual production.

### 4.4. Combined Use and Analysis of GC–MS and E–Nose

GC–MS and E–nose were used and analysed in combination to establish associations between sensors and specific flavour substances. Seven sensors, including W5S, W6S, W1S, W1W, W2S, W2W and W3S, were positively correlated with 10 key flavour compounds (1–nonanol, 3–methyl–1–butanol acetate, hexanoic acid ethyl ester, heptanoic acid ethyl ester, octanoic acid ethyl ester, decanoic acid ethyl ester, dodecanoic acid ethyl ester, benzenepropanoic acid ethyl ester, furfural and 1–(2,6,6–trimethyl–1,3–cyclohexadien–1–yl)–2–buten–1–one), indicating that the seven sensors can effectively reflect the strong aroma of SW and DL.

The association of GC–MS and E–nose needs to be an effective method to detect food flavour, considering that the detection accuracy of GC–MS is high, but it takes a long time, while E–nose detection is fast, but it cannot determine the specific substance. Huang et al. [60] integrated the volatile compounds and electronic nose response values of sugarcane juice under different treatments, and took Euclidean distance as the metric standard to generate the cluster analysis heat map of each index, and found that the response value of S6 may be related to 1–amyl alcohol and 1–octene–3–alcohol, and the response value of S8 and S10 may be related to the content of ethanethiol to a certain extent. Wu et al. [61] established the correlation between volatile compounds in the different dried *Chrysanthemum nankingense* and the response value of the E–nose sensor. Sensor W1C had a good correlation with C62 (hexyl n–valerate), sensor W5C had a good correlation with C136 (phenanthrene), and sensor W3C had a close correlation with C109 (geranialene). The sensor W1W had high sensitivity to alkenes, and had good correlation with various terpenes, such as C117 (caryophyllene) and C130 (1–isopropyl–4, 7–dimethyl–1,2,3,5,6,8 a–hexahydronaphthalene).

## 5. Conclusions

In this study, changes in the flavour of SW and DL at different processing stages were compared, the differential metabolites were analysed, and relationships between E–nose sensor values and key flavour compounds were established. A total of 133 VOCs were detected in the three groups, with 76, 79 and 99 VOCs identified in SJ, SW and DL, respectively. Sixteen and 17 VOCs contributed to the flavour of SW and DL, respectively. Fermentation significantly increased the contents of VOCs, especially esters. 3–Octanone and phenylethyl alcohol were the most significantly upregulated VOCs. E–nose data showed that seven sensors could reflect the increases in contents of alcohols, esters and 10 key flavour compounds.

## Figures and Tables

**Table 1:** OAV of odour–active components in SJ, SW and DL by GC–MS (*n* = 3).

No.	Compounds	Threshold (μg/kg)	Content (mg/L)			OAV		
			SJ	SW	DL	SJ	SW	DL
al9	1–Octanol	54.000	0.098 ± 0.050	0.026 ± 0.002	0.070 ± 0.008	1.8	0.5	1.3
al12	1–Nonanol	2.000	0.120 ± 0.072	0.067 ± 0.005	0.306 ± 0.042	60.0	33.3	152.8
al17	Phenylethyl alcohol	45.000	0.448 ± 0.132	0.936 ± 0.118	––	10.0	20.8	––
e1	3–Methyl–butanoic acid ethyl ester	6.890	0.129 ± 0.006	0.387 ± 0.018	0.067 ± 0.007	18.7	56.2	9.7
e2	Pentanoic acid ethyl ester	94.000	0.153 ± 0.010	0.458 ± 0.029	0.123 ± 0.030	1.6	4.9	1.3
e3	3–Methyl–1–butanol acetate	3.000	––	––	0.118 ± 0.022	––	––	39.3
e11	Hexanoic acid ethyl ester	0.500	0.103 ± 0.001	0.309 ± 0.003	0.335 ± 0.005	206.0	618.0	670.0
e14	Heptanoic acid ethyl ester	170.000	0.067 ± 0.020	0.201 ± 0.060	0.270 ± 0.053	0.4	1.2	1.6
e19	Octanoic acid ethyl ester	0.100	0.137 ± 0.038	0.411 ± 0.114	1.647 ± 0.348	1370.0	4110.0	16,466.7
e21	3–Methyl–butanoic acid hexyl ester	0.150	0.040 ± 0.019	0.120 ± 0.057	0.036 ± 0.010	266.7	800.0	240.0
e27	Decanoic acid ethyl ester	20.000	––	––	1.233 ± 0.336	––	––	61.7
e37	Benzeneacetic acid ethyl ester	100.000	0.087 ± 0.048	0.261 ± 0.044	0.143 ± 0.019	0.9	2.6	1.4
e40	Dodecanoic acid ethyl ester	330.000	––	––	1.110 ± 0.276	––	––	3.4
e44	Benzenepropanoic acid ethyl ester	0.060	––	––	0.029 ± 0.004	––	––	488.9
c4	Heptanal	10.000	0.005 ± 0.001	0.015 ± 0.002	––	0.5	1.5	––
c6	Nonanal	15.000	0.088 ± 0.035	0.264 ± 0.105	0.052 ± 0.012	5.9	17.6	3.5
c8	Furfural	100.000	0.034 ± 0.003	0.102 ± 0.009	0.444 ± 0.090	0.3	1.0	4.4
c10	5–Methyl–2–furancarboxaldehyde	50.000	0.053 ± 0.011	0.159 ± 0.053	0.015 ± 0.001	1.1	3.2	0.3
c13	Tetradecanal	60.000	0.097 ± 0.009	0.284 ± 0.069	––	1.6	4.7	––
c14	6,10–dimethyl–5,9–Undecadien–2–one	100.000	0.056 ± 0.013	0.169 ± 0.058	––	0.5	1.7	––
c15	1–(2,6,6–Trimethyl–1,3–cyclohexadien–1–yl)–2–buten–1–one	0.001	––	––	0.133 ± 0.029	––	––	132,666.7
o3	4–Ethylphenol	10.000	0.085 ± 0.012	0.254 ± 0.045	––	8.4	25.4	––

## Data Availability

Data is contained within the article and supplementary material.

## Supplementary Materials

The following supporting information can be downloaded at: https://www.mdpi.com/article/10.3390/foods11203273/s1. Figure S1: Comparison of pathways in SJ, SW and DL by GC–MS (*n* = 3). (A) Main pathways by metabolites annotation, and log2FC (B) and VIP (C) for differential pathways after fermentation; Figure S2: Butanoate metabolism; Figure S3: Furfural degradation; Table S1: Concentration, p and VIP of VOCs of SJ, SW and DL.
