# Supplementary material for: Novel Insight into the Formation of Odour—Active Compounds in Sea Buckthorn Wine and Distilled Liquor Based on GC–MS and E–Nose Analysis
Source: Foods. 2022 Oct 20;11(20):3273. doi: 10.3390/foods11203273 (PMC9601902; doi:10.3390/foods11203273)
Supplement: Supplementary file 1 [file foods-11-03273-s001.zip › foods-1953054-supplementary.pdf]

Table S1. Concentration, *p* and VIP of VOCs of SJ, SW and DL.

No.	Compound	Juice	Wine	Distillate	*p*	VIP
al1	2-Butanol	0.1243±0.019	0.101 ±0.015	0.074 ±0.012	0.521	0.283
al2	2-methyl-Propanol	--	0.095 ±0.011	0.115 ±0.015	0.316	0.418
al3	3-methyl-Butanol	0.137±0.137	--	1.463 ±0.193	0.150	2.363
al4	1-Hexanol	0.033±0.018	0.012 ±0.002	0.004 ±0.001	0.004	0.204
al5	3-ethoxy-1-Propanol	--	0.024 ±0.000	0.030 ±0.002	0.047	0.192
al6	1-Heptanol	--	0.012 ±0.002	0.019 ±0.004	0.322	0.155
al7	Linalool	0.082±0.029	0.040 ±0.012	0.333 ±0.013	0.790	1.141
al8	[S-(R*,R*)]-2,3-Butanediol	--	0.259 ±0.145	--	0.129	1.308
al9	1-Octanol	0.098±0.050	0.026 ±0.002	0.070 ±0.008	0.001	0.542
al10	[R-(R*,R*)]-2,3-Butanediol	0.004±0.001	0.085 ±0.015	--	0.131	0.699
al11	Terpinen-4-ol	0.053±0.004	0.020 ±0.000	0.038 ±0.006	0.000	0.311
al12	1-Nonanol	0.120±0.072	0.067 ±0.005	0.306 ±0.042	0.123	1.044
al13	2-Furanmethanol	0.077±0.017	0.021 ±0.001	--	0.000	0.318
al14	α-Terpineol	--	--	0.057 ±0.008	0.109	0.472
al15	Geraniol	--	--	0.014 ±0.005	0.119	0.249
al16	Benzyl alcohol	--	0.054 ±0.002	--	0.022	0.643
al17	Phenylethyl Alcohol	0.448±0.132	0.936 ±0.118	--	0.154	2.415
e1	3-methyl-Butanoic acid ethyl ester	0.129±0.006	0.387 ±0.018	0.067 ±0.007	0.227	1.528
e2	Pentanoic acid ethyl ester	0.153±0.010	0.458 ±0.029	0.123 ±0.030	0.506	1.583
e3	3-methyl-1-Butanol acetate	--	--	0.118 ±0.022	0.132	0.678
e4	Butanoic acid, 1-methylpropyl ester	0.041±0.002	0.124 ±0.005	--	0.046	0.921
e5	Pentanoic acid 1-methylpropyl ester	0.408±0.005	1.224 ±0.015	1.380 ±0.361	0.142	1.175
e6	2-methyl-Butanoic acid 2-methylpropyl ester	0.013±0.001	0.039 ±0.003	0.044 ±0.006	0.194	0.204
e7	Isobutyl isovalerate	0.072±0.001	0.216 ±0.002	0.622 ±0.057	0.131	1.081
e8	2-methyl-Propanoic acid 2-methylbutyl ester	0.035±0.001	0.103 ±0.002	0.250 ±0.067	0.137	0.631
e9	Pentanoic acid 1-methylpropyl ester	--	--	0.659 ±0.049	0.131	1.690
e10	Hexanoic acid 1-methylethyl ester	0.033±0.001	0.099 ±0.003	--	0.164	0.822
e11	Hexanoic acid ethyl ester	0.103±0.001	0.309 ±0.003	0.335 ±0.005	0.134	0.749

e12	2-methyl-Butanoic acid 2-methylbutyl ester	0.507±0.009	1.521 ±0.027	0.835 ±0.026	0.155	2.407
e13	3-methyl-Butanoic acid 2-methylbutyl ester	2.297±0.017	6.889 ±0.050	4.477 ±1.012	0.145	4.635
e14	Heptanoic acid ethyl ester	0.067±0.020	0.201 ±0.060	0.270 ±0.053	0.543	0.382
e15	Hexanoic acid 2-methylpropyl ester	--	--	0.030 ±0.004	0.132	0.339
e16	3-methyl-Butanoic acid 3-methyl-3-butenyl ester	--	--	0.035 ±0.006	0.101	0.372
e17	Octanoic acid methyl ester	0.025±0.002	0.075 ±0.016	0.021 ±0.003	0.180	0.553
e18	n-Octanoic acid isopropyl ester	0.056±0.016	0.168 ±0.078	--	0.164	1.020
e19	Octanoic acid ethyl ester	0.137±0.038	0.411 ±0.114	1.647 ±0.348	0.259	1.950
e20	2-methyl-Propanoic acid heptyl ester	0.016±0.001	0.048 ±0.013	--	0.164	0.529
e21	3-methyl-Butanoic acid hexyl ester	0.040±0.019	0.120 ±0.057	0.036 ±0.010	0.240	0.731
e22	Hexanoic acid 2-methylbutyl ester	0.102±0.099	0.306 ±0.107	0.186 ±0.034	0.209	0.869
e23	(Z)-4-Octenoic acid ethyl ester	0.041±0.022	0.124 ±0.065	--	0.153	0.869
e24	Nonanoic acid, ethyl ester	0.039±0.024	0.117 ±0.032	0.272 ±0.063	0.543	0.582
e25	3-hydroxy-Butanoic acid ethyl ester	0.029±0.024	0.086 ±0.011	--	0.155	0.698
e26	2-Furancarboxylic acid 1-methylethyl ester	0.052±0.044	0.156 ±0.032	--	0.164	0.938
e27	Decanoic acid ethyl ester	--	--	1.233 ±0.336	0.132	2.171
e28	Benzoic acid 1-methylethyl ester	0.068±0.042	0.205 ±0.025	0.120 ±0.017	0.212	0.722
e29	Benzoic acid ethyl ester	0.076±0.049	0.227 ±0.046	0.445 ±0.027	0.238	0.648
e30	3-methyl-Butanoic acid 2-furanylmethyl ester	0.070±0.045	0.210 ±0.035	0.065 ±0.004	0.191	0.929
e31	2-Furanecarboxylic acid 2-butyl ester	0.116±0.058	0.348 ±0.174	0.119 ±0.017	0.201	1.199
e32	Undecanoic acid ethyl ester	--	--	0.035 ±0.004	0.132	0.368
e33	Benzoic acid 2-methylpropyl ester	0.153±0.146	0.458 ±0.037	--	0.162	1.595
e34	Benzoic acid cyclohexyl ester	--	--	1.006 ±0.151	0.130	1.987
e35	n-Propyl benzoate	0.058±0.002	0.174 ±0.056	0.030 ±0.001	0.166	0.900
e36	Methyl salicylate	0.028±0.026	0.083 ±0.017	0.029 ±0.004	0.173	0.552
e37	Benzeneacetic acid ethyl ester	0.087±0.048	0.261 ±0.044	0.143 ±0.019	0.217	0.857
e38	Benzoic acid 2-methylpropyl ester	0.148±0.110	0.445 ±0.029	0.220 ±0.039	0.209	1.152
e39	Acetic acid 2-phenylethyl ester	0.067±0.063	0.202 ±0.088	0.244 ±0.034	0.237	0.432
e40	Dodecanoic acid ethyl ester	--	--	1.110 ±0.276	0.132	2.065

e41	2-Furancarboxylic acid 3-methylbutyl ester	0.065±0.002	0.195 ±0.086	0.045 ±0.018	0.201	0.919
e42	Hexanoic acid 2-phenylethyl ester	0.088±0.012	0.264 ±0.016	--	0.164	1.224
e43	β-Phenylethyl butyrate	--	--	0.105 ±0.008	0.132	0.640
e44	Benzenepropanoic acid ethyl ester	--	--	0.029 ±0.004	0.106	0.337
e45	3-methyl-Butanoic acid phenylmethyl ester	0.107±0.088	0.321 ±0.064	--	0.164	1.349
e46	Pentanoic acid phenylmethyl ester	--	--	0.254 ±0.044	0.132	0.996
e47	3-methyl-Butanol benzoate	0.099±0.060	0.296 ±0.079	1.315 ±0.205	0.515	1.780
e48	Ethyl tridecanoate	--	--	0.031 ±0.008	0.109	0.361
e49	3-methyl-Butanoic acid 2-phenylethyl ester	0.103±0.094	0.310 ±0.081	0.236 ±0.044	0.227	0.751
e50	phenyl-Acetic acid isopentyl ester	--	--	0.024 ±0.003	0.072	0.310
e51	Methyl tetradecanoate	--	--	0.021 ±0.008	0.132	0.280
e52	Tetradecanoic acid ethyl ester	0.072±0.009	0.216 ±0.007	0.795 ±0.155	0.528	1.268
e53	Isoamyl laurate	--	--	0.020 ±0.005	0.095	0.290
e54	Ethyl 9-tetradecenoate	--	--	0.050 ±0.011	0.132	0.440
e55	3-phenyl-2-Propenoic acid ethyl ester	--	--	0.021 ±0.009	0.132	0.279
e56	Pentadecanoic acid ethyl ester	--	--	0.043 ±0.012	0.132	0.405
e57	Hexadecanoic acid methyl ester	--	--	0.038 ±0.004	0.086	0.387
e58	9-Hexadecenoic acid methyl ester	--	--	0.044 ±0.006	0.132	0.411
e59	Hexadecanoic acid ethyl ester	0.075±0.014	0.225 ±0.022	1.145 ±0.230	0.534	1.690
e60	Hexadecanoic acid propyl ester	--	--	0.015 ±0.002	0.047	0.241
e61	Ethyl 9,12-hexadecadienoate	--	--	0.039 ±0.007	0.106	0.389
e62	Ethyl Oleate	--	--	0.058 ±0.017	0.132	0.461
e63	Linoleic acid ethyl ester	--	--	0.049 ±0.006	0.132	0.440
ac1	Acetic acid	0.260±0.109	0.176 ±0.045	--	0.002	0.756
ac2	2-methyl-Propanoic acid	--	--	0.080 ±0.022	0.132	0.553
ac3	3-methyl-Butanoic acid	0.047±0.017	0.057 ±0.003	0.174 ±0.050	0.198	0.593
ac4	Hexanoic acid	0.088±0.024	0.021 ±0.002	0.035 ±0.008	0.000	0.375
ac5	Octanoic acid	0.163±0.160	0.037 ±0.006	0.056 ±0.013	0.000	0.502
ac6	Oxalacetic acid	0.124±0.019	--	--	0.111	0.478
ac7	Nonanoic acid	--	--	0.019 ±0.005	0.132	0.270
ac8	n-Decanoic acid	0.123±0.024	--	--	0.127	0.475
ac9	Dodecanoic acid	--	--	0.042 ±0.002	0.047	0.409

ac10	Benzoic acid	0.091±0.056	--	--	0.020	0.424
ac11	n-Hexadecanoic acid	--	0.041 ±0.016	0.091 ±0.013	0.304	0.449
c1	Hexanal	--	--	0.002 ±0.000	0.132	0.083
c2	4-methylene-5-Hexenal	--	--	0.012 ±0.003	0.132	0.215
c3	3-Octanone	0.011±0.002	0.034 ±0.005	0.013 ±0.001	0.000	0.402
c4	Heptanal	0.005±0.001	0.015 ±0.002	--	0.138	0.292
c5	6-methyl-5-Hepten-2-one	0.176±0.061	0.527 ±0.182	0.043 ±0.004	0.143	1.838
c6	Nonanal	0.088±0.035	0.264 ±0.105	0.052 ±0.012	0.401	1.178
c7	1-(1H-pyrazol-4-yl)-Ethanone	0.097±0.014	0.291 ±0.022	--	0.138	1.292
c8	Furfural	0.034±0.003	0.102 ±0.009	0.444 ±0.090	0.475	1.009
c9	Benzaldehyde	0.086±0.036	0.257 ±0.017	0.077 ±0.013	0.427	1.053
c10	5-methyl-2-Furancarboxaldehyde	0.053±0.011	0.159 ±0.053	0.015 ±0.001	0.141	0.895
c11	4-dimethyl-3-Cyclohexene-1-acetaldehyde	--	--	0.045 ±0.007	0.079	0.420
c12	2-Tridecanone	--	--	0.021 ±0.003	0.038	0.290
c13	Tetradecanal	0.097±0.009	0.284 ±0.069	--	0.136	1.256
c14	6,10-dimethyl-5,9-Undecadien-2-one	0.056±0.013	0.169 ±0.058	--	0.135	0.970
c15	1-(2,6,6-trimethyl-1,3-cyclohexadien-1-yl)-2-Buten-1-one	--	--	0.133 ±0.029	0.074	0.717
c16	Pentadecanal	--	--	0.023 ±0.006	0.079	0.297
c17	1-(4-Methoxymethyl-2,6-dimethylphenyl)ethanone	--	--	0.024 ±0.006	0.057	0.302
c18	Hexadecanal	--	--	0.023 ±0.005	0.079	0.298
c19	2,3-dihydro-3,5-dihydroxy-6-methyl-4H-Pyran-4-one	0.087±0.088	0.262 ±0.063	--	0.136	1.202
c20	5-Hydroxymethylfurfural	0.499±0.093	1.499 ±0.278	--	0.138	2.879
alk1	3,7-dimethyl-1,3,7-Octatriene	0.001±0.000	--	--	0.022	0.049
alk2	1,1-dimethyl-Cyclopropane	--	0.495 ±0.084	--	0.079	1.702
alk3	Tridecane	0.041±0.001	--	--	0.079	0.288
alk4	Styrene	--	0.015 ±0.001	--	0.079	0.341
alk5	3-ethyl-Tridecane	--	0.008 ±0.001	--	0.047	0.256
alk6	Tetradecane	0.024±0.017	0.001 ±0.000	0.013 ±0.003	0.233	0.280
alk7	5-Tetradecene	0.026±0.002	--	--	0.079	0.223
alk8	3,5-dimethyl-Cyclohexene	--	--	0.022 ±0.005	0.079	0.291

alk9	Pentadecane	0.039±0.005	0.003 ±0.001	--	0.091	0.263
alk10	4-methylene-1-(1-methylethyl)-Cyclohexene	--	0.011 ±0.002	--	0.000	0.293
alk11	nonyl-Cyclopropane	--	--	0.020 ±0.008	0.062	0.276
t1	β-copaene	0.037±0.003	--	--	0.074	0.264
t2	Germacrene D	--	0.019 ±0.001	0.030 ±0.002	0.085	0.226
t3	α-Muurolene	0.027±0.002	--	--	0.079	0.227
t4	α-Farnesene	--	--	0.021 ±0.005	0.079	0.277
t5	Selina-3,7(11)-diene	--	--	0.009 ±0.010	0.065	0.179
t6	Dispiro [4.2.4.2]tetradeca-6,13-diene	--	--	0.007 ±0.001	0.060	0.151
t7	Cadina-1(10),6,8-triene	--	--	0.021 ±0.004	0.079	0.268
t8	α-Copaene	--	--	0.005 ±0.001	0.079	0.131
o1	2-methoxy-Phenol	0.066±0.006	0.197 ±0.079	--	0.135	1.049
o2	Phenol	0.071±0.006	0.213 ±0.010	--	0.138	1.085
o3	4-ethyl-Phenol	0.085±0.012	0.254 ±0.045	--	0.136	1.187
o4	2-Methoxy-4-vinylphenol	--	--	0.017 ±0.003	0.079	0.258
o5	2,4-Di-tert-butylphenol	--	--	0.037 ±0.005	0.053	0.384
o6	trans-Isoeugenol	--	--	0.025 ±0.002	0.079	0.315
o7	2-methoxy-4-(1-propenyl)-Phenol	0.119±0.117	0.359 ±0.050	--	0.137	1.410
o8	Acetoin	0.080±0.029	0.240 ±0.087	--	0.138	1.237
o9	(2S,4R)-4-Methyl-2-(2-methylprop-1-en-1-yl)tetrahydro-2H-pyran	--	--	0.042 ±0.005	0.053	0.406
o10	1-ethenyl-4-methoxy-Benzene	--	--	0.030 ±0.006	0.057	0.344
o11	2,3-dihydro-1,1,5,6-tetramethyl-1H-Indene	--	--	0.020 ±0.002	0.079	0.261
o12	(+)-Borneol	--	--	0.092 ±0.018	0.079	0.584
o13	methoxy-phenyl-Oxime	0.114±0.076	0.341 ±0.027	0.081 ±0.018	0.165	1.235
o14	1, 1, 5-Trimethyl-1, 2-dihydronaphthalene	--	--	0.008 ±0.001	0.004	0.177
o15	2,3-dihydro-Benzofuran	--	--	0.021 ±0.002	0.022	0.295

Note: VIP (Variable important in Projection) was the variable weight value of the (O)PLS-DA model variables (SIMCA-P software), which can be used to measure the im-pact of different metabolite accumulation differences on the classification and discrimination of each group of samples and the explanatory power. VIP ≥ 1 was common screening criterion for different metabolites.

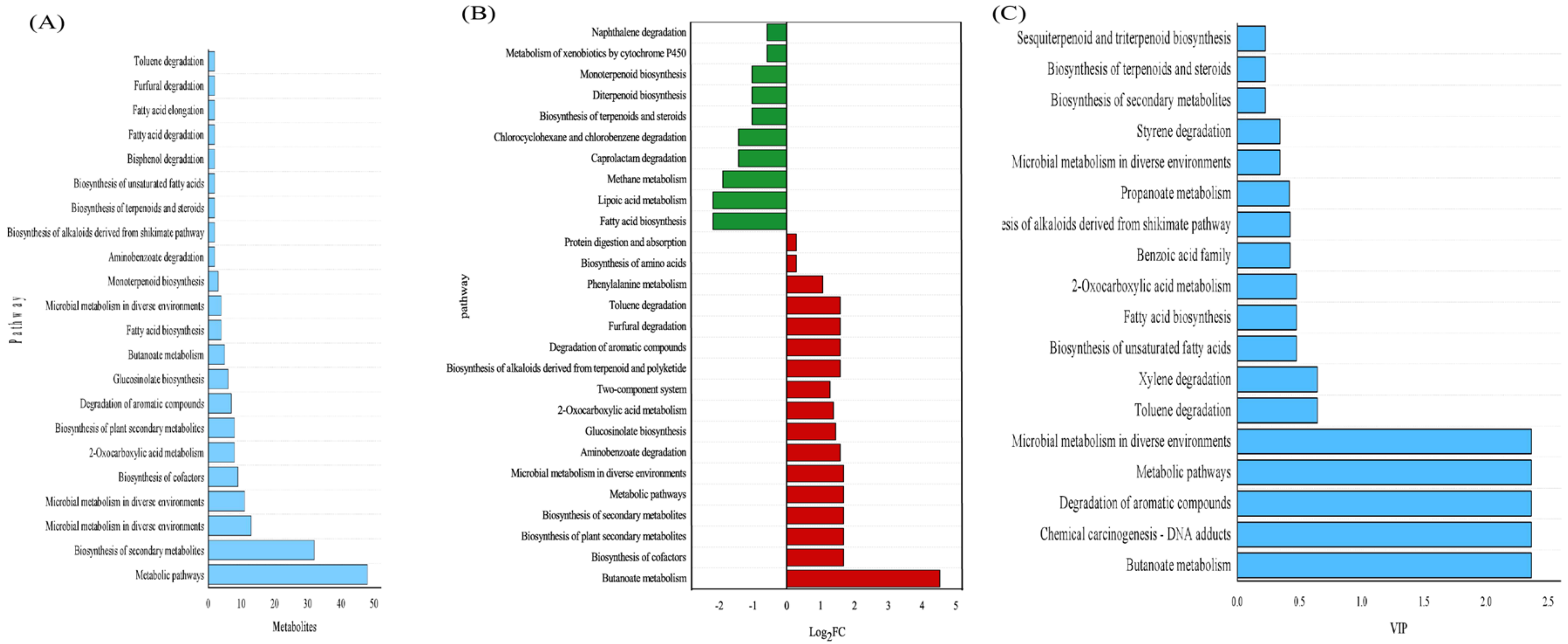

Figure S1. Comparison of pathways in SJ and SW by GC-MS (**n** = 3). (**A**) Main pathways by metabolites annotation, Log2FC (**B**) and VIP (**C**) for differential pathways after fermentation.

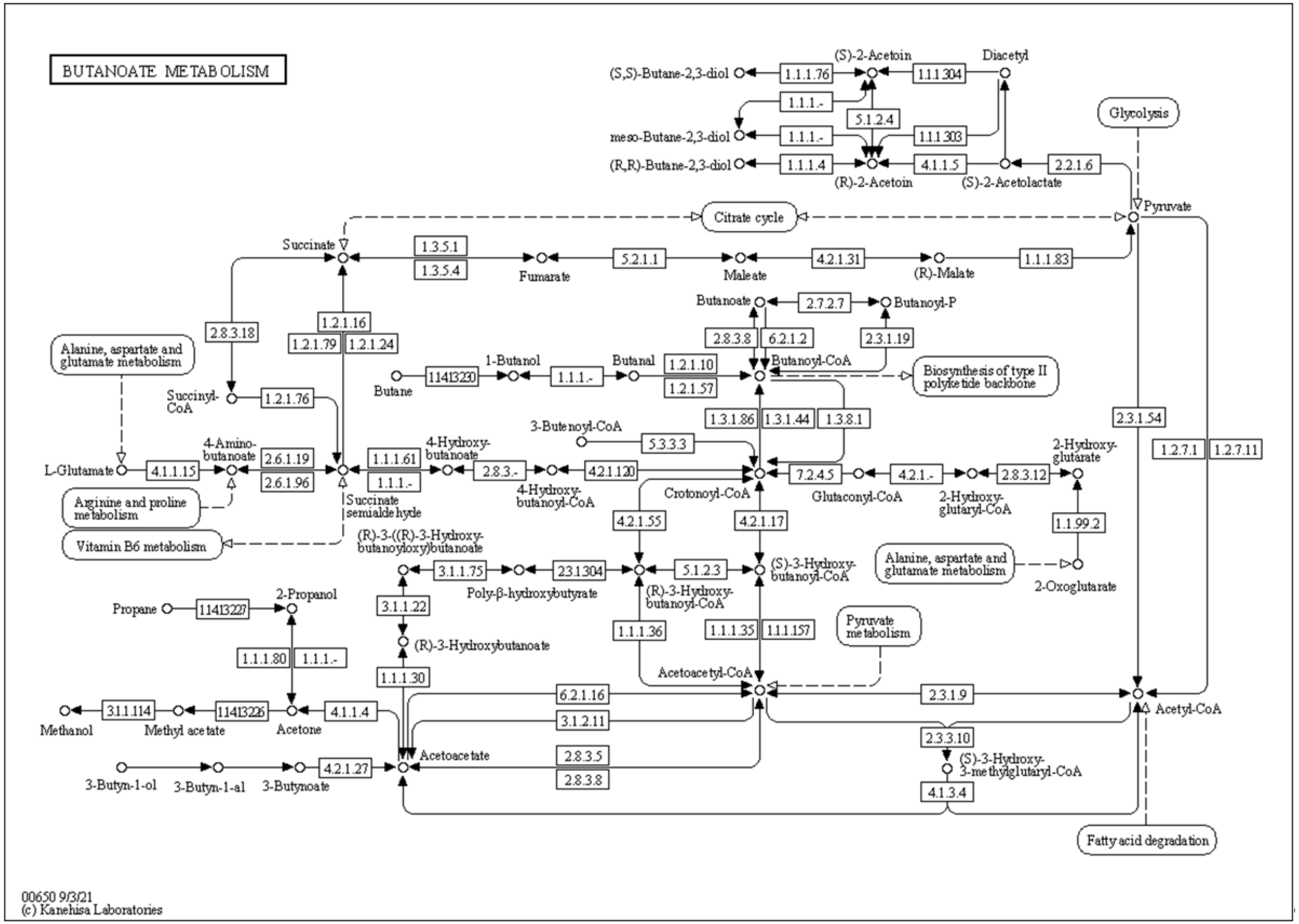

Figure S2. Butanoate Metabolism.

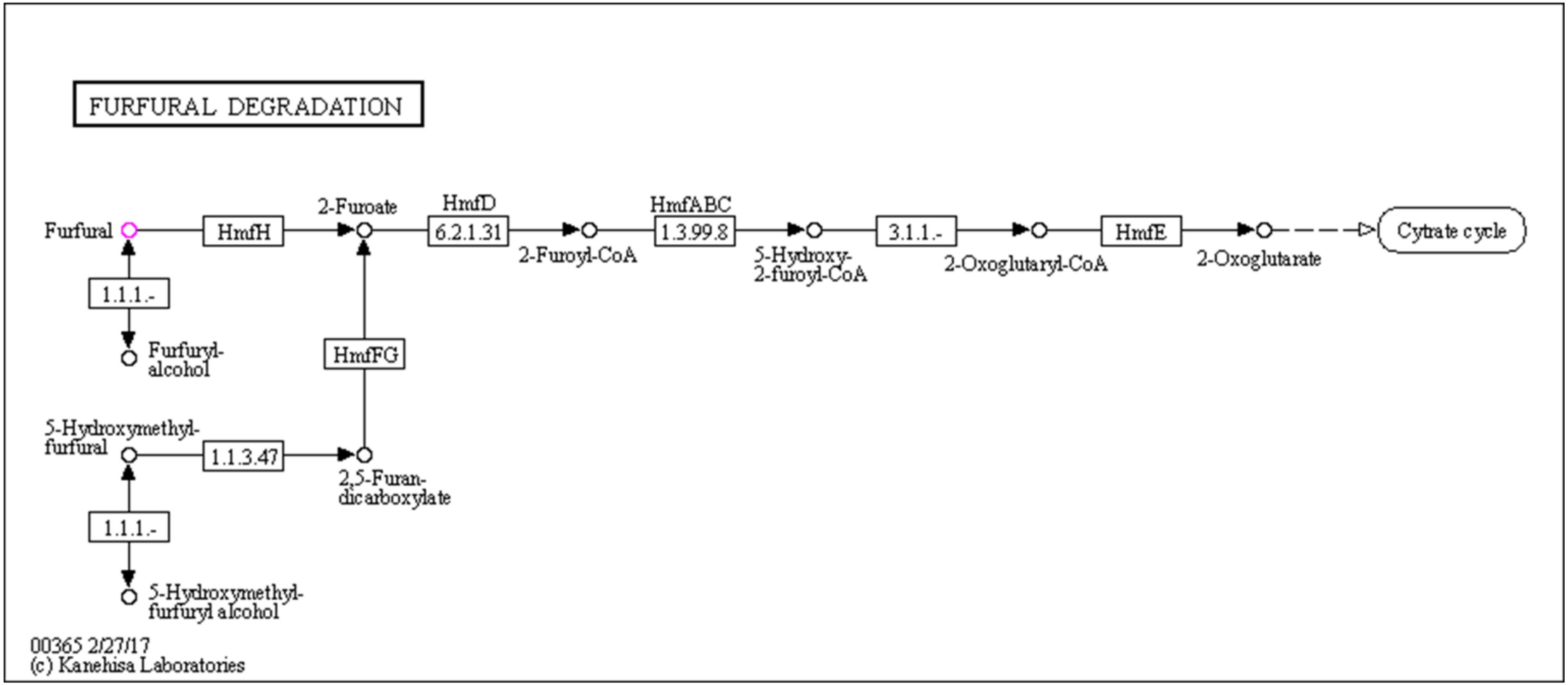

Figure S3. Furfural degradation.
